# Standing next to but not being part of: relatives’ experiences of support from healthcare professionals when general palliative care is provided at home

**DOI:** 10.1186/s12904-026-02021-3

**Published:** 2026-02-13

**Authors:** Elina Mikaelsson Midlöv, Therese Sterner, Susann Porter, Terese Lindberg, Katarina Sjögren Forss

**Affiliations:** 1 Blekinge Institute of Technology, Faculty of Engineering, Department of Health, Valhallavägen 1, Karlskrona, 371 79 Sweden; 2 Faculty of Health and Society, Department of Care Science, Malmö University, Malmö, Sweden

**Keywords:** Home care, Palliative care, Relatives, Support, Support needs

## Abstract

**Background:**

Relatives play a crucial role when palliative care is provided at home. More advanced care at home places higher demands on relatives, taking great responsibility, facing challenges, and often lacking adequate knowledge and skills to provide care. Therefore, relatives need support from healthcare professionals, yet do not receive the needed support. This study aimed to elucidate relatives’ experiences of support from healthcare professionals before and after a patient’s death when general palliative care is provided at home.

**Methods:**

A phenomenological hermeneutical method was used. The inclusion criteria were relatives of people who had died, involved in general palliative care at home. The sample consisted of 14 adult relatives involved in general palliative care at home between one week and 12 months. Data were collected through individual interviews between January and May 2025.

**Results:**

Relatives needed to be seen as they felt left out; they felt an overwhelming responsibility; they needed to feel safe at home through guidance from and access to healthcare professionals; they felt the need to know what was happening and what to expect; and they needed help in processing the grief both before and after the patient’s death. These themes formed the main theme: Standing next to but not being part of.

**Conclusions:**

The findings of this study showed a lack of support for relatives before and after the patient’s death but offer insights into what support relatives need from HCPs when general PC is provided at home. Relatives need to feel seen, informed and prepared, to feel safe when care is provided at home, and not feel overwhelmed by the responsibility of the situation. As research continuously reveals that relatives have unmet support needs, this highlights the need for tailored interventions and the targeting of available support actions for improved support. Since relatives play a crucial role in palliative care at home, continued work with education and training for relatives should be prioritised to support them in feeling prepared, obtaining necessary caregiving knowledge and skills, enabling them to cope with the situation at home.

**Supplementary Information:**

The online version contains supplementary material available at 10.1186/s12904-026-02021-3.

## Background

The provision of palliative care (PC) at home has become increasingly common, and relatives, such as family or friends, often play a crucial role in the caregiving process [1–3]. With more advanced care at home, this places higher demands on relatives [2, 4, 5], such as symptom control, medications, heavy lifting and management of advanced medical equipment. The responsibilities taken by relatives vary from providing companionship, emotionally supporting the patient, performing all household chores, to medical tasks, coordinating care and making difficult decisions regarding the patient. Nevertheless, when PC is provided at home, it often relies on the efforts of relatives, who take great responsibility, face challenges and often lack adequate knowledge and skills to provide care, thus putting them at risk of ill health themselves [2, 4, 6–9]. These health risks may manifest as emotional stress, physical exhaustion, insomnia or depression. Relatives may also experience isolation, feelings of fear, anxiety, hopelessness, disempowerment and guilt in the situation [7, 8, 10, 11]. Therefore, relatives need support from healthcare professionals (HCPs) when PC is provided at home, both during the period of care and after a patient’s death. Supporting relatives may include HCPs assisting relatives in the care and offering informational, emotional and practical support. Education, information, personalised relief and various forms of guidance and counselling are essential [5], aiming to prevent ill health and enhance coping capacity before and after a patient’s death [12].

In Sweden, healthcare responsibility is divided between 21 regions and 290 municipalities. While regions are responsible for specialised healthcare, municipalities are responsible for general healthcare [13], which includes healthcare services at home, including general PC. PC includes general and specialised forms, and of the 90,000 who die each year in Sweden, 70,000 are assessed as needing PC, of whom 22,000 are estimated to need specialised PC. However, access and quality of general and specialist PC vary geographically [14]. In home care, general PC is mainly provided. General PC is usually provided by HCPs with basic PC knowledge without it being their main activity [14], primarily by registered nurses (RNs) and nursing assistants (NAs), with RNs having a leading role [13]. In Sweden, HCPs include licensed professionals, such as RNs, but also unlicensed professionals, such as NAs, as they assist the licensed personnel in the care of patients [15]. As municipalities do not employ doctors, RNs are highly medically responsible and available around the clock, but with limited access during evenings, nights and weekends when covering larger areas. When needed, RNs contact regional primary care doctors. If general PC is not sufficient to meet the needs of patients and relatives, specialised PC, with the region responsible, is added [14]. So, general PC at home, provided by HCPs in the municipalities, can be supplemented with specialised PC at home, and then also include a multi-professional team of HCPs, usually with a higher level of education and a main focus on providing PC, including doctors [14].

Both home care and PC have evolved over the years, and in Sweden, national guidelines and knowledge support for PC have been developed, providing guidance and recommendations to support healthcare providers in developing and providing equal PC, including training recommendations for HCPs [12, 14]. Despite these advances, challenges persist, such as HCPs’ competence and training and support for relatives [16]. Even though support for relatives is emphasised in PC [17, 18], previous research has shown that those involved in PC at home have unmet support needs before and after a patient’s death [10, 19–24], and that the support provided by HCPs is deficient [19, 23, 25, 26]. These unmet needs—defined as the absence of desirable or necessary actions or resources that support well-being [27]—highlight the importance of tailored support to meet the needs that a person cannot maintain on their own. Relatives’ support needs can change quickly in PC, depending on the situation and the patient’s condition [16, 28], making support challenging, especially in general PC at home [14, 16], where resources and expertise may be limited. Although there is extensive research on support for relatives in PC, support for relatives in general PC at home is less described from the relatives’ perspective, and research after a patient’s death is sparse. To improve support when general PC is provided at home, further research is needed about relatives’ specific needs, as it can provide valuable insights to better support them. Therefore, this study aimed to elucidate relatives’ experiences of support from HCPs before and after a patient’s death when general PC is provided at home.

## Methods

### Design

This study used a phenomenological hermeneutical method guided by Lindseth and Norberg [29] and inspired by Ricoeur [30], which focuses on the participants’ lived experiences, chosen to gain an understanding of the deeper meaning of these experiences [29]. The authors followed the Consolidated Criteria for Reporting Qualitative Research checklist [31] for explicit reporting for improved transparency and study quality (see Additional File 1).

### Participants and setting

The inclusion criteria for this study were adult relatives of adult persons who had died, and had been involved in general PC at home, with care provided by HCPs from the municipality. No upper limit was set for years passed since being part of general PC at home, to not restrict access to potential participants, as the chosen method [29] focuses on elucidating the experiences of relatives regardless of when they occurred. Variations were sought in terms of gender, age and type of relative. The sample consisted of 14 relatives (12 women and 2 men) aged 32–85 years who were either a spouse, partner, child, parent or sibling of the deceased, and all had been informal carers, some more, some less. The participants were relatives of deceased persons aged 25–94 years, evenly distributed between men and women, who had died of various forms of cancer, stroke, chronic obstructive pulmonary disease, myasthenia gravis, dementia, and kidney failure. One participant was a relative of two deceased persons who died within a short period of time. They had been involved in general PC at home, with home care provided by HCPs (RNs and NAs) between one week and up to 12 months, in seven different municipalities in four counties in southern Sweden. In addition to general PC, three of the participants had also received help from HCPs from specialised PC teams. See Table 1 for the participants’ demographics and characteristics.

**Table 1:** Overview of the participants’ demographics and characteristics (*n* = 14)

Gender	Age	Type of relative	Care provided at home by HCPs	Death occurred at home	Years since death	Municipality
Female (12)	32 (1)	Spouse (5)	1 week by RN in home care (3)	Yes (8)	0.5 (1)	Urban (13)
Male (2)	33 (1)	Partner (1)	A few weeks by RN and NA in home care (1)	No (6)	2 (3)	Rural (1)
	40 (1)	Child (6)	2 months by RN and NA in home care (2)		2.5 (4)	
	48 (1)	Parent (1)	3 months by RN in home care (1)		3 (1)	
	49 (1)	Sibling (1)	5 months by RN and NA in home care (1)		5 (1)	
	61 (1)		6 months by RN and NA in home care (1)		6.5 (1)	
	67 (2)		A few months by RN in home care (2)		10 (1)	
	70 (2)		12 months by RN in home care (1)		12 (1)	
	72 (2)		12 months by RN and NA in home care (2)		17 (1)	
	73 (1)					
	85 (1)					

*HCPs* Healthcare professionals, *NA* Nursing assistant, *PC* Palliative care, *RN* Registered nurse

### Recruitment and data collection

To recruit participants, information sheets about the study were distributed physically at Blekinge Institute of Technology’s research clinic (a transdisciplinary research environment where academia, industry, regions and municipalities collaborate and where national and international clinical research projects are conducted) and digitally on the first author’s social media platforms, Facebook and LinkedIn. The information sheets explained the study’s purpose, stated that participation was voluntary and could be ended at any time without giving a reason, and included the research team’s contact details and information on who was conducting the study. Those interested in participating contacted the first author via phone or e-mail. These relatives received further oral and written study information, were given the opportunity to ask questions and provided consent to participate. In total, 14 participants expressed interest in participating, and since all of them met the study’s inclusion criteria, no one was excluded from participation. Data were collected from January to May 2025 through individual interviews. A pilot interview was conducted by the first author to evaluate the questions. No changes were needed because the answers that emerged were in accordance with the aim of the study, and the interview was therefore included in the study. In total, 14 interviews were conducted by the first author. The participants chose how and where the interviews would take place. Twelve of the interviews were conducted face-to-face, four in the participants’ homes and eight in a neutral meeting room at Blekinge Institute of Technology. Two of the interviews were conducted digitally using Microsoft Teams and Zoom.

Before each interview began, questions were asked to confirm that participants had been part of general PC at home, with HCPs from the municipality. The interviews started with the open question, ‘Tell us how your relative came to receive general PC at home’, and the participants were encouraged to talk freely about their experience in their own words. A semi-structured interview guide was used as a reminder to ensure that both the support before and after the patient’s death was covered and to obtain detailed narratives (Table 2). Follow-up questions were asked to encourage deepening. Supporting notes regarding impressions and thoughts were written during and directly after the interviews. Throughout and at the end of each interview, the interviewer confirmed the participants’ stories by summarising what they shared to ensure that their stories were collected and not interpreted. The interviews were conducted in Swedish, and each interview lasted 55–150 min, with an average time of 92 min. After 14 interviews, the data collection was finalised. These interviews provided detailed and informative narratives, creating rich data material, and were considered a preferred number to achieve depth and variety in experiences [32].

**Table 2:** Description of the questions in the interview guide

Questions in the interview guide
Tell us how your relative came to receive general PC at home.
Tell us about your experience of the support you received from HCPs during the period of care at home.
Tell us if there is anything you wish had been different about the support you received from HCPs during the period of care at home.
Tell us about your experience of the support you received from HCPs at the time of your relative’s death.
Tell us if there is anything you wish had been different about the support you received from HCPs at the time of your relative’s death.
Tell us about your experience of the support you received from HCPs after your relative’s death.
Tell us if there is anything you wish had been different about the support you received from HCPs after your relative’s death.

### Data analysis

All interviews were audio-recorded on a dictaphone, and the first author transcribed the material verbatim. The analysis was based on 14 interviews (of which one covered two cases), resulting in 287 pages of transcribed interview material in a Microsoft Word document. The analysis was conducted in Swedish to preserve the original meanings. After the analysis and completion of the results section, the findings and quotes were translated into English. A phenomenological hermeneutical analysis was conducted in three steps: naïve reading, structural analysis and comprehensive understanding [29]. First, the interviews were read several times with an open mind to grasp the meaning, and a naïve understanding was formulated. A thematic structural analysis was then conducted. The text was read and viewed as objectively as possible and divided into meaning units. Similarities and differences were reflected on, and the meaning units were condensed and then abstracted to form themes. The themes were reflected on in relation to the naïve understanding, which was validated by the structural analysis. Finally, the text was open mindedly read again as a whole, with both the naïve understanding and the themes in mind, and then interpreted based on pre-understandings and relevant literature to reach a comprehensive understanding. The interpretation process was inspired by Ricoeur [30], moving between the parts and the whole of the text to reach an understanding through explanation.

An awareness of how professional and personal experiences and pre-understanding influence the process was evident throughout, to remain open to the data and participants’ narratives, with reflexivity being central in the analysis. Lindseth and Norberg [29], however, describe the importance of the researcher having sufficient pre-understanding to grasp the essential meaning of the text. This means that the first author’s clinical experience as an RN in general PC for a few years was beneficial in the analysis process. The first author conducted a preliminary analysis and discussed it first with the last author and then with the other authors. Dialogues were conducted until a consensus was reached.

## Results

### Naïve understanding

The relatives described the period of care at home as stressful, and they felt that too much responsibility was placed on them, which they perceived as unmanageable without the support of HCPs. They described needing guidance from and access to HCPs during the period of care at home and after the patient’s death, and emphasised the importance of being prepared, knowing what is happening, getting clear information and having ongoing communication with HCPs. Although some support was offered, it was perceived as insufficient and sometimes non-existent, leading to feelings of anxiety, helplessness, loneliness and being left out. The relatives described how they felt overlooked by HCPs who focused primarily on the patient. They described the need to be noticed by HCPs and for them to see their need for support, as the relatives put their own needs aside and adapted to meet the needs of the dying person, which often led to exhaustion. The relatives felt the need to be listened to and supported by HCPs in processing their experiences of what they had been going through.

### Structural analysis

**Fig. 1:**
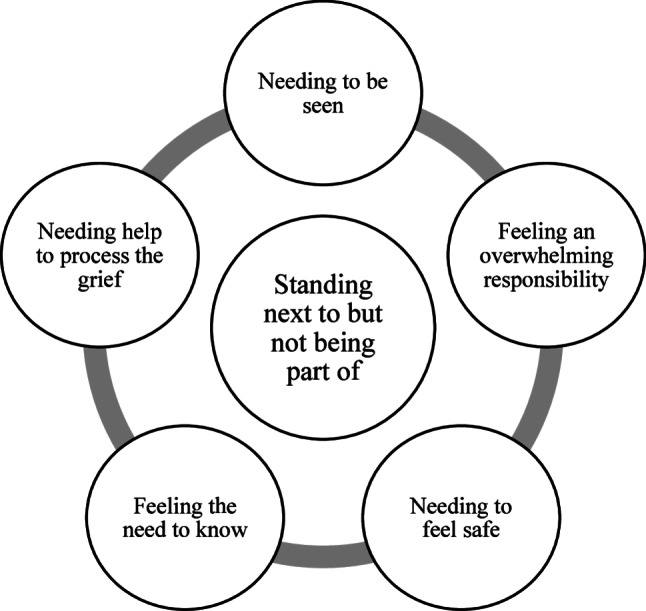
Overview of the themes

The relatives’ experiences of support from HCPs in general PC at home are elucidated through five themes: needing to be seen, feeling an overwhelming responsibility, needing to feel safe, feeling the need to know and needing help to process the grief. These form the foundation of the main theme: Standing next to but not being part of (Fig. 1).

### Needing to be seen

The relatives described the need to feel seen by HCPs both during the period of care at home and after the patient’s death. They felt left out, as the focus remained solely on the patient, despite their significant role. The HCPs did not ask about their willingness to provide care at home, even though their efforts were crucial. Many relatives took on substantial responsibilities but were unable to ease their own burdens, lacking adequate support from HCPs. While some relatives felt seen and received support, most did not, feeling unnoticed and unsupported.

*I can say that they didn’t notice ME during that time. Or us. They didn’t. No one did. No one asked how WE were doing. I didn’t get any support. I probably would’ve had to seek it out myself*,* but no one noticed us and asked how we were doing. (P4)*

Furthermore, the relatives who worked during this time described feeling left out when it came to financial aspects, as they did not receive help from doctors in obtaining sick leave to care for the patient. As only one relative at a time was entitled to financial compensation, others wishing to be present during the final phase were excluded from this support. Several relatives were unaware of the available support options, which made them feel unseen as HCPs did not inform them. They described feeling lonely during the period of care at home, often left alone with the patient and without support from HCPs. Many felt that they had no one to contact for help and expressed the desire to be noticed by HCPs and supported throughout the period of care and after the patient’s death.

*It wasn’t so much about me…There was no network for me. No one noticed me*,* so I had to know what I needed and ask for that help myself. Otherwise*,* I don’t think I would have gotten as much for myself. (P1)*

Some relatives felt seen by HCPs through regular check-ins, questions about their well-being, and clear communication, and reported a more positive experience of the care period at home. Some relatives also felt seen by HCPs when their caregiving efforts were acknowledged, their emotional needs validated and their need for relief recognised and addressed.

*I appreciated that it wasn’t just about my sister*,* even though it obviously was*,* but we were also part of it. (P6)*

Furthermore, most relatives described feeling excluded and unsupported by HCPs after the patient’s death. They would have liked the HCPs to recognise their needs and reach out to them afterwards. The sudden absence of contact, following an intense period of caregiving and interaction with the HCPs, left them feeling alone, unseen and not part of this.

*Afterwards*,* it felt so empty. Everyone disappeared… It was only about my husband*,* just as I had understood all along. (P2)*

### Feeling an overwhelming responsibility

The relatives felt that an overwhelming responsibility was placed on them when care was provided at home and that the HCPs relied on them too much. This responsibility made many relatives experience the period as intense and stressful, finding themselves in situations they had never experienced before or imagined. Despite feeling overwhelmed, they supported the patient’s wish to be cared for and die at home.

I was completely unhappy. I didn’t know what to do. (P13)

Several relatives felt that they needed more support from the HCPs to avoid feeling overwhelmed by the responsibility of caring for the patient at home, but they still struggled on their own. They described being left with tasks they felt unprepared for, such as administering injections when the HCPs did not have time to come. Despite this, they performed tasks to avoid patient suffering. Those relatives wished that the HCPs had asked whether they were comfortable performing such tasks, rather than assuming their willingness. Furthermore, some relatives experienced an overwhelming responsibility as they were constantly busy with caregiving tasks, such as watching over the patient, administering medication, assisting with hygiene and managing equipment, often without rest. The lack of sleep and continuous demands significantly affected their resilience. Some relatives requested nighttime relief from NAs to sleep, but such support was rarely provided. Several of the relatives described managing all this alongside their own family and work responsibilities, which were often neglected. In retrospect, they questioned how they had managed to do it all.

*But it didn’t really work out well in the end. We couldn’t take it anymore. It could have worked well if we had been able to sleep. (P3)*

The relatives described having to adapt to the dying person’s needs, often feeling like an overwhelming responsibility. Many assumed a caregiving role that prioritised the patient while neglecting their own needs. In addition to caregiving at home, several relatives took responsibility for managing tasks such as collecting medications, consumables, and aids and contacting various healthcare institutions. When multiple institutions were involved, coordination issues arose, giving relatives more responsibility to prevent errors. They wished that the HCPs had recognised their burden and informed them that more support was available to prevent them from feeling like they had to manage everything by themselves.

*No one said*,* ‘This must be difficult for you. Let’s do it this way instead’. It was difficult… The HCPs knew that we were there a lot. In hindsight*,* perhaps if they had been a little clearer about it so that we could have thought about ourselves a little more*,* things might have been a little different. (P8)*

Furthermore, the relatives felt helpless when pain relief at home was ineffective. Even after contacting them, the HCPs would either not show up or be delayed. Some relatives had to take responsibility and manage the situation themselves. In desperation, some administered medication without authorisation, while others were unable to act, resulting in patient suffering. Several relatives described the final days as a terrible experience, as they felt frustrated and helpless due to poor pain relief and inadequate support and attention from HCPs.

*They just tell us that they can’t make it*,* so we must try to manage it on our own. She (the RN) puts all these injections on the kitchen counter with a note saying that we should give them every four hours and so on. It was me and our two daughters who were at home. And we kept doing this through the night on Friday*,* the night on Saturday and the night on Sunday*,* when she died. It was awful. But we felt we had no other choice. We were left completely on our own those days*,* from when they said they couldn’t come until the night she died. No one came here all weekend. When you really needed someone*,* some kind of support and help with all this*,* you didn’t get it. (P5)*

However, some relatives described receiving excellent support from HCPs in coping with their responsibilities, which made the situation at home feel manageable. They appreciated being offered relief when needed and having their responsibilities acknowledged. Thus, they were allowed brief moments for themselves, even though they remained worried about the patient in their absence.

*You felt that they supported you. Because I felt—how lonely I would have been otherwise if I hadn’t had them to support me. So it was great. (P13)*

Furthermore, several relatives experienced physical and psychological health issues during this period, due to the burden and feeling of an overwhelming responsibility. In several cases, they felt that the situation at home eventually became untenable because they were completely exhausted and could no longer handle the responsibility, necessitating the patient’s transfer to a healthcare institution to die. They appreciated it when the HCPs noticed it, sparing them from having to initiate this decision themselves.

*That day*,* they decided to take him in so that I could rest. I washed and hung out the sheets because he was sweating so much. Then I brought the sheets in and thought I had to make the bed too*,* and then I fell into the bed. So I kind of spun around*,* and then I tried again to put the sheet on*,* and then I fell again. I was so tired that I couldn’t get it together. (P13)*

### Needing to feel safe

The relatives described the need to feel safe with HCPs in home care. They felt safe and supported when HCPs were easily accessible. All relatives had 24/7 phone access to RNs, providing safety because they could get advice and help when needed. However, the various response times and the HCPs arriving or not arriving led to safety concerns among the relatives. Despite easy accessibility by phone, technical issues sometimes hindered contact, and some relatives were unable to reach HCPs at critical moments, such as at the time of the patient’s death, which compromised their sense of safety.

*We had telephone contact. It was easy to reach them*,* and they came when we needed them. (P1)*

The relatives’ need to feel safe with easy accessibility to HCPs was influenced by their own social networks, education and knowledge of PC. Even those with support from family and friends or who had healthcare training themselves expressed the need for support from HCPs, although the need seemed to be less. When the relatives themselves were RNs and felt safe performing certain medical tasks, their need for HCP support was reduced, but they emphasised the importance of having their willingness confirmed rather than assumed. Other relatives, despite being HCPs themselves, preferred to remain in the role of a relative, as they did not feel comfortable performing certain tasks, having to actively communicate this to the HCPs.

*I said that I don’t do that. Now I’m just a wife*,* so I don’t take care of things. I don’t flush the drain. I don’t do anything except help him to the bathroom*,* make the bed*,* and stuff like that. But I don’t do anything else. (P7)*

Furthermore, the relatives described feeling unsafe if something were to happen at night and during weekends, as the HCPs were not as available then, and they wished for improved preparation, routines and support during these times. Many also emphasised the need for better preparedness for pain relief in the home during these times.

*The saddest thing of all was that the last days of her life were not good. Not at all… Extremely frustrating. It was unfortunate that it happened precisely on the weekend. If it had been a weekday*,* she would probably have received help in a different way. She would have been able to pass away more peacefully. We thought it was incredibly sad that she had such a painful death. It’s just that better preparedness is needed when things happen on the weekend. They didn’t give her medicine over the weekend because they didn’t have a prescription for it*,* and the district doctor wasn’t available. She also wasn’t registered in that group (specialised PC teams)*,* so they couldn’t contact those doctors. (P3)*

Some relatives described that HCPs would make preventive home visits, thus reducing their need to call for support. Knowing that HCPs would come contributed to a sense of safety. While many lacked continuity among HCPs, some of the relatives felt safe having continuity among HCPs in the home, as it led to trust and improved understanding of their support needs. The relatives emphasised the importance of HCPs taking the time to stay with them, showing personal engagement or offering extra support, because it created a sense of safety.

*I would say that the important thing for me was that it was the same HCPs—that was probably what meant the most to us. Because it became a bit like*,* ‘since yesterday*,* what has happened’? You didn’t have to go back much. They became like family members in a way. I think that support was the best thing… A commitment. They didn’t just come and do what they had to do. (P9)*

However, several relatives perceived the HCPs, especially the NAs, to be impersonal and focused solely on minimal patient care tasks with no interest in the relatives. Sometimes, the NAs did not show up at all, contributing to a sense of insecurity among the relatives.

*The NAs were not sufficient to provide PC*,* but I don’t blame them for that. They are certainly doing their best*,* but it’s not easy. Perhaps the visits or other things were missed because there was no continuity*,* new people arrived*,* and so on. (P11)*

Some relatives also expressed a desire to change the HCPs they felt uncomfortable with, uncertain whether this was possible. Holiday periods further heightened several relatives’ feelings of insecurity, as unfamiliar HCPs would often arrive.

*We didn’t get along at all… It feels better if you trust them… that’s important. (P14)*

According to the relatives, the competence of HCPs influenced their feelings of safety at home. While few concerns were raised regarding the competence of RNs, many described issues with NAs, including insufficient competence and frequent communication failures, which contributed to the relatives’ feelings of unsafety.

*One of the first nights that this NA watched over her*,* she developed a pressure ulcer on her lower back. We had taken care of her for six weeks without her getting the slightest (pressure ulcer)… but it’s this ignorance. These guys sat there with their phones all night long. If Mom had gotten help turning over at night*,* she would have avoided this pressure ulcer. (P3)*

Furthermore, several relatives wanted more structured support and described how it would have felt safe to have HCPs continuously checking their support needs during the period of care at home and for a period after the patient’s death. Some relatives also wanted more access to doctors and counsellors at home as they were required to contact primary or specialist care if the need arose.

*Some kind of support throughout the whole process*,* like someone calling me once a week or having appointments booked in advance… Just being caught up… and I probably would’ve needed some support afterward*,* for a limited time. To have it as standard—that you’re followed up for three months*,* once a month*,* or something. Or if you want more conversations or have someone to contact. I think that would have been great. (P1)*

Many relatives wanted guidance from HCPs in navigating the care system, including assistance with phone calls and contacting other HCPs and healthcare institutions. They described the feeling of uncertainty about roles and responsibilities, combined with the burden of managing communication themselves, as time-and energy-consuming. Thus, they would have appreciated help—such as a coach—throughout the whole situation.

*It was tough because you had to be some kind of project manager. Everything is so complicated… You just think*,* please*,* can’t you just do this… Suddenly*,* there are so many people at different clinics and in different places. So*,* sometimes*,* you don’t always know who is responsible for what and where to turn to for certain things. (P10)*

Most relatives appreciated the presence of HCPs at the time of death because it provided a sense of safety, as they helped prepare the patient, explained the process and answered their questions.

### Feeling the need to know

The relatives emphasised the need for informative support from HCPs throughout the period of care at home and after the patient’s death. They needed to know what was happening and what to expect, including details about the illness, physical changes and the dying process, so they could be prepared. They considered clear information, explanations and justifications for treatments or medications from HCPs important, as many were often uncertain about the reasons behind care decisions.

*Information is extremely important. That you are guided in some way and that they ensure that you’ll get all the information that is available. We wanted more information about the disease itself. What can we expect from now on? It’s not easy to prepare for that. We were not given any information about that. That’s what we would have liked to have had a little more of. What could happen and what would we do in that case… If we had been told that she was going to die*,* we could’ve done things differently… Made the most of her last days in a different way. (P5)*

Those relatives who also received support from HCPs in specialised PC reported receiving clear and sufficient information about what to expect from them before and after the patient’s death. In contrast, others described receiving insufficient or no information at all, leaving them feeling unprepared and alone at home. They emphasised the importance of receiving clear and understandable information and communication from HCPs. Some felt that communication was unclear, particularly regarding the proximity of death, thus leaving them unprepared for how little time remained. While some acknowledged that denial could have influenced their understanding, they also experienced difficulty processing information during this period. Several relatives also noted the lack of structure in how and what information was communicated by HCPs, which contributed to their confusion and uncertainty.

*I think it would have been better if they had a better structure… Now we gather everyone together*,* we have conversations*,* and everyone gets the same information… (P12)*.

Most relatives described that the information from HCPs about practical aids available to them at home, such as beds, wheelchairs and toilet seats, as well as information about financial compensation for caring for a closely related person, was clear and helpful. Some were also satisfied with the informative support they received at the time of the patient’s death, as they received verbal and written information about what was happening and what to expect going forward.

### Needing help to process the grief

The relatives described needing help in processing their grief and needing someone to talk to outside the family about the situation during the period of care at home and after the patient’s death. Many appreciated it when HCPs offered counselling or facilitated contact with a counsellor, as they found it difficult to take the initiative. However, some relatives reported that they were never offered such support. In contrast, those relatives who also had help from HCPs in specialised PC were offered counselling through them. Those who received counselling from a counsellor found it helpful in managing their grief and overall situation, and they also received guidance on how to communicate with the patient and children in the family. The relatives who needed counselling but did not receive it found it difficult to cope with their grief and expressed a strong desire for a professional to talk with.

*I had no one to talk to about the situation. I really wanted someone (professional) I could just sit and cry with. I couldn’t do that with my daughters either*,* because they had enough of their own problems. I didn’t want to burden them with it. (P5)*

For many relatives, part of the grieving process was the opportunity to have final conversations with HCPs a few weeks after the patient’s death. However, several reported not being offered such conversations and noted a complete lack of contact with HCPs after the patient’s death. Those few who were offered final conversations with HCPs found it essential to express their emotions and receive answers to lingering questions. This support was mainly offered to those who had also received help from HCPs in specialised PC. Furthermore, several relatives described needing professional counselling to process their grief and the experiences surrounding the patient’s death. However, many found it difficult to seek that support on their own and preferred to be given that support from HCPs.

*I probably needed something*,* a few conversations. Just knowing that you had two conversations so you could*,* ‘Oh yes*,* I forgot to ask about that’*,* and so on. That they came back… It would have felt good to have closure. That would have been desirable because it would have probably helped me let go of a lot of the things that I was thinking and worrying about. It would have probably cleared things up so that I could let go and move on much faster or find balance afterwards. Because it’s not just like cutting a thread or something. There are a lot of aftereffects. (P13)*

Several relatives also wanted to say goodbye to the RNs and NAs who had been with them at home, describing this as a meaningful part of their grieving process. While most were not given this opportunity, those who were emphasised its value.

*What I miss most is not being able to say goodbye to everyone we had*,* even the RNs we had in the evenings and at night. I still feel a little sad about that. (P2)*

### Comprehensive understanding

The relatives’ experiences of support from HCPs before and after the patient’s death when general PC was provided at home consist of five connecting themes. The findings are interpreted as that relatives’ experiences can be understood from the main theme: ‘standing next to but not being part of’. The care period at home was interpreted as being characterised as emotional and stressful, with relatives facing situations that they could not handle themselves, thus leading them to need support, to which HCPs must be responsive. When support was not provided, the relatives felt alone, left out, helpless and ignored by HCPs, which could intensify a feeling of standing next to but not being part of the care at home. When they were not seen by HCPs and did not know what to do or expect, the burden of responsibility they felt increased, leaving them feeling alone and emotionally exhausted. Being seen and knowing what to do were crucial for the relatives to be prepared, cope with the overwhelming responsibilities that often arise and process grief. Adequate and clear information and communication with HCPs were emphasised and necessary for the relatives to feel safe at home and to navigate through the care period at home and after the patient’s death. Standing next to but not being part of illustrates that relatives experienced that they were excluded from care during a transition in their lives that many found difficult to cope with. This transition, as described by Melei et al. [33], involves changes in roles, relationships, and health that arise, for example, in connection with illness and death, and was evident in relatives’ narratives of a passage to a situation dominated by illness and imminent death. Their need to be seen, feel safe, to know and be informed, and to need help to process the grief both before and after the patient’s death, reflects the challenges of navigating this transition and striving for a sense of normality. Understanding the experiences of relatives through transition theory highlights why their support needs vary over time, and over the two care periods. Relatives in PC often experience a range of transitions, from the time before diagnosis to the time after death, which can be challenging and stressful, and they seek normality throughout transitions [34, 35]. Relatives may need support to manage these transitions in a constructive way, and HCPs have an important role in facilitating the transition [33–36]. An awareness of these transitions can form the basis for supportive phase-specific interventions [34–36] that help relatives feel prepared and maintain control during the care period at home and after the patient’s death.

## Discussion

The findings from this study show that relatives need support from HCPs throughout the period of care at home and after the patient’s death, as they experienced an overwhelming responsibility and faced situations difficult to handle by themselves. They experienced that they needed to feel seen, know and be prepared, to feel safe at home and not feel an overwhelming responsibility due to the situation. When not being seen by HCPs and not receiving adequate support, many relatives felt lonely and helpless, also describing putting aside their own needs. This is also evident in other studies [37–39], showing that relatives often neglect their own needs when PC is provided at home, which can negatively affect their well-being [40, 41], even though caring for the patient may also bring positive experiences [42]. Although relatives want HCPs to be aware of their well-being during the period of care at home and after the patient’s death, HCPs rarely notice their well-being [39]. Relatives’ experiences of needing to be seen can be understood as a desire for recognition and being acknowledged as partners in care [38]. If HCPs do not have a dual focus and see relatives, noticing their well-being and need for support, in a situation which can be difficult to cope with on your own, this can increase emotional strain and undermine their ability in managing care at home. The relatives in this study experienced that they needed to know what was happening and what to expect, including details about the illness, physical changes and the dying process, to be prepared for the situation at home. However, several relatives described unmet information needs and wanted more communication and informational support from HCPs. Previous studies have emphasised the importance of relatives being prepared to handle care at home, with relatives reporting feeling prepared when they receive clear and honest information about the prognosis of the illness and the changes to be expected; knowledge about medication, treatment and care; information about death and the process of dying; and knowing whom to contact 24/7 depending on the situation [38, 41]. Obtaining adequate information helps relatives manage care responsibilities, cope with uncertainty and prepare for the end of life and the grieving process [39]. The findings in this study also showed that many relatives experienced helplessness due to the lack of informational support and attention from HCPs regarding pain relief at home. Some felt alone with an overwhelming responsibility, managing the situation by themselves. This is in accordance with previous research describing relatives experiencing challenges in managing the patient’s symptoms and needing more support [41, 43]. Relatives struggle with medication knowledge, such as recognising and responding to symptoms (e.g. pain). Nevertheless, they administer medication to the patient without having received any training [44] and want practical training and guidance in pain management [38]. When relatives are responsible for assessing and managing symptoms, they need support in knowing what to monitor, how to interpret symptoms and when to contact HCPs; otherwise, the burden may be perceived as unmanageable [22]. Relatives’ experiences of needing to know show the importance of informational support, influencing relatives’ sense of control in a situation characterised by uncertainty. If HCPs do not meet these needs, emotional strain may increase and managing care at home may become more challenging. Therefore, adequate information can help relatives feel more prepared for the situation when general PC is provided at home, reducing the perceived burden and its negative impact on well-being.

As relatives experienced that they needed to know more, and many also felt an overwhelming responsibility, HCPs could further support relatives in being prepared to handle PC at home and acquiring the necessary caregiving knowledge by offering them training courses. This has also been suggested in previous studies [38, 41, 45]. Relatives often need education and training to acquire the knowledge, skills and resources that will enable them to manage PC at home and increase the possibility of better well-being and care experience. Three primary learning needs in PC have been identified for relatives: understanding the illness and its progression, mastering the required caregiving skills and knowing how to access support from HCPs [46]. Therefore, education must include information that leads to knowledge and understanding about the illness and its progression, symptoms and symptom management, the dying process, medication and administration, equipment management and practical caregiving skills and techniques to perform tasks safely [41]. As a complement to receiving this from HCPs in person, previous studies have demonstrated the importance of using online learning resources for education, benefiting relatives’ support needs [47, 48]. In specialised PC, web-based interventions have been carried out, for example, a psychoeducational intervention in Sweden preparing relatives for the patient’s progressing illness and death through a website with short videos supplemented by informative texts, a chat forum and web links [48]. Due to limited healthcare resources, web-based support may be a useful alternative to in-person interventions to meet the support needs of relatives [48]. However, while targeted education and training could help address relatives’ need to know and, to some extent, reduce their feeling of an overwhelming responsibility, by improving their feeling of being informed and prepared for PC at home, this must be considered in relation to the other themes identified in the results. It must be balanced with support that addresses emotional strain, the need to be seen, and feelings of exclusion, still making relational and emotional support essential, both before and after the patient’s death.

The findings in this study further showed that many relatives experienced feeling safe and supported when HCPs were easily accessible, even though they also described being unable to reach HCPs when needed. Feeling safe with care at home is fundamental for relatives’ ability to cope with the situation and not feel left alone in critical situations. Needing accessibility to HCPs is evident in previous research [23, 39, 49], and their absence can place a burden on relatives, as they may lack the necessary knowledge and information to cope with the overwhelming situation at home [49]. As PC at home means an extended staff absence, relatives need to be prepared for it through guidance, knowledge, tools and accessible support from HCPs to feel that they can handle the situation at home during the HCPs’ absence [50]. Based on relatives’ experiences in this study, improving accessibility to HCPs is crucial, especially when resources are limited, e.g., at night and on weekends. One way to achieve this could be through digital solutions. Digital technology could optimise the use of healthcare resources and access to HCPs. For instance, internet-based PC consultations can facilitate timely information, guidance, and communication with HCPs, making HCPs more accessible to relatives [43, 51]. This could also apply to accessing HCPs and getting extended support for relatives after the patient’s death to help process their grief, as multiple relatives in the study experienced a sudden absence of contact with HCPs after the patient’s death, leaving them feeling alone.

Furthermore, the findings showed that several relatives experienced wanting more structured support from HCPs based on their needs when general PC is provided at home. The desire for structured support may reflect relatives’ need to feel guided by HCPs to reduce the feeling of an overwhelming responsibility. However, it is evident in previous studies that there is no systematic approach when it comes to supporting relatives [19, 25, 26]. HCPs must apply structured ways of offering support to relatives to increase the chances of meeting their support needs, both before and after a patient’s death. According to the findings in this study, some relatives described that one way of offering structured support is to have a care coordinator guiding them through the care system and facilitating contact with other HCPs and healthcare institutions. The importance of relieving relatives from this coordinating role has also been emphasised in other studies [52]. As coordinating PC at home involves numerous challenges for relatives, a designated care coordinator can help them feel safe and better cope with the situation [49, 52]. Thus, further research on general PC at home could focus on how access to a care coordinator could affect relatives’ experience of coping with providing PC at home, and the possibility of reducing the caregiver burden and increasing the feeling of being supported.

When PC is provided at home, the support provided by HCPs affects relatives’ ability to cope with the situation and their well-being. Therefore, HCPs need to be more responsive to and meet the changing support needs of relatives during the care period at home and after the patient’s death, especially in general PC at home, in which resources and expertise may be limited. This study contributes to the field by elucidating relatives’ experiences of support from HCPs when general PC is provided at home and capturing the experience of support needs both before and after a patient’s death, extending existing knowledge. The findings and the main theme, standing next to but not being part of, contribute to a deeper understanding of why HCPs need to adopt a dual focus and actively support relatives, rather than just assuming their involvement. The study’s findings provide insights that can form the basis for strategies and guide practical interventions to better meet relatives’ needs.

### Methodological considerations

Although the study aimed to elucidate relatives’ experiences of support from HCPs in general PC, three participants, in addition to receiving support from HCPs providing general PC, also received support from HCPs in specialist PC teams. The interviews focused on general PC, and distinctions between HCPs in home care and specialist teams were clear in relatives’ narratives. However, three descriptions in the results mention specialised PC, included as they clarify what these relatives experienced was lacking in general PC, directly relevant to the aim. Other aspects of specialised PC were excluded from the analysis. This approach ensured that the interpretation remained focused on general PC while acknowledging relatives’ comparisons as part of their lived experience. The sample was predominantly female, which may reflect the fact that more women than men tend to assume the role of informal carers [53, 54]. However, this may have influenced the results as previous research shows that women tend to be more involved in care and psychologically strained [53, 54], and the experiences of male relatives may be underrepresented, potentially affecting transferability. Furthermore, participants were included regardless of how much time had passed since their involvement in general PC at home, as the method [29] focuses on elucidating the experiences of relatives regardless of when they occurred. This resulted in not excluding relevant experiences and ethically inclusive participation, not denying interested relatives the opportunity to participate and share their narratives. Even participants for whom several years had passed shared detailed narratives about their experiences. However, the variation in time since the participants were involved in general PC at home means that the interviews were based on retrospective memories, which could influence how the participants interpreted and recounted their experiences, as experiences can change or be reevaluated. This could have influenced the results, also considering that PC and home care have developed during this time. However, similar experiences were recounted regarding the support provided by HCPs, regardless of how much time had passed. The participants also considered the passing of time an advantage, as it gave them time to reflect on what support they needed before and after the patient’s death. Furthermore, the duration of involvement in general PC at home, with home care provided by HCPs, varied among participants, which may have influenced their experiences. However, both those who received care for a shorter period and those with longer care periods described similar experiences and challenges, suggesting that these were not solely dependent on care length, although it may have had some impact. Therefore, the duration of care is not explicitly presented in the results section. Throughout the analysis process, critical reflection and discussion took place among the authors to ensure the trustworthiness of the interpretation and the credibility of the findings. An advantage of the analysis process is the dialectic movement between the whole and the parts, and comparing the naïve understanding, the structural analysis and the comprehensive understanding may ensure trustworthiness in the analysis process [29]. According to Ricoeur [30], a text is not limited to one meaning and can be interpreted in more than one proper way, but all the authors agreed that this was a credible understanding of the narratives.

## Conclusion

To increase the possibility of meeting relatives’ support needs, it is important to understand and address how they experience the support provided by HCPs at home. The findings of this study show a lack of support to relatives before and after the patient’s death but offer insights into what support relatives need from HCPs when general PC is provided at home. The findings can benefit both relatives and HCPs in clinical practice, but also inform policymakers in developing structured support for relatives. Relatives need to be seen, informed and prepared to feel safe when care is provided at home, and not feel overwhelmed by the responsibility of the situation. They also need extended support after the patient’s death to help process their grief. Furthermore, this study confirms that the support provided by HCPs influences relatives’ ability to cope with the situation at home, and this highlights the need for tailored interventions and the targeting of available support actions for improved support. As relatives play a crucial role in PC at home, and research continuously reveals how relatives have unmet support needs when general PC is provided at home, further research in this area is important. Continued work with education and training for relatives should be prioritised to support them in feeling prepared, obtaining the necessary caregiving knowledge and skills, enabling them to cope with the situation at home, and reducing the burdensome feeling. Further research on general PC at home could therefore focus on web-based interventions that enhance relatives’ preparedness and caregiving competence, as well as on access to digital care coordinators and their potential to relieve relatives’ coordinating responsibilities and caregiver burden. Digital access to HCPs could also be part of the support to help relatives process the grief after a patient’s death.

## Supplementary Information

Supplementary Material 1.

## Data Availability

The data that support the findings of this study are not publicly available due to privacy or ethical restrictions but are available on reasonable request from the corresponding author.

## Abbreviations

HCPs: HealthCare Professionals
NAs: Nursing Assistants
PC: Palliative Care
RNs: Registered Nurses
