# Supplementary material for: Standing next to but not being part of: relatives’ experiences of support from healthcare professionals when general palliative care is provided at home
Source: BMC Palliat Care. 2026 Feb 13;25:54. doi: 10.1186/s12904-026-02021-3 (PMC12930732; doi:10.1186/s12904-026-02021-3)
Supplement: Supplementary file 1 — Supplementary Material 1. [file 12904_2026_2021_MOESM1_ESM.pdf]

Additional file 1. The Consolidated Criteria for Reporting Qualitative studies (COREQ): 32-item checklist

No	Item	Guide question/description	
**Domain 1: Research team and reflexivity**			
Personal Characteristics			
1.	Interviewer/facilitator	Which author/s conducted the interview or focus group?	EMM conducted the interviews.
2.	Credentials	What were the researcher's credentials? *E.g. PhD, MD*	EMM: PhD student<br>SP: PhD<br>TS: PhD<br>KSF: PhD<br>TL: PhD
3.	Occupation	What was their occupation at the time of the study?	EMM: University lecturer<br>SP: Associate Senior Lecturer<br>TS: Senior Lecturer<br>KSF: Associate professor/ Senior Lecturer<br>TL: Senior Lecturer
4.	Gender	Was the researcher male or female?	Female
5.	Experience and training	What experience or training did the researcher have?	EMM: Nursing<br>SP: Health Science<br>TS: Health Science/ Nursing<br>KSF: Public health and health science<br>TL: Health Science/ Nursing<br><br>All researchers had experience in qualitative methodology and interview methods.
Relationship with participants			
6.	Relationship established	Was a relationship established prior to study commencement?	No
7.	Participant knowledge of the interviewer	What did the participants know about the researcher? *e.g. personal goals, reasons for doing the research*	They knew the reasons for doing the research and the affiliations of the researcher.
8.	Interviewer characteristics	What characteristics were reported about the interviewer/facilitator? *e.g. Bias, assumptions, reasons and interests in the research topic*	Reasons and interests in the research topic.
**Domain 2: study design**			
Theoretical framework			
9.	Methodological orientation and Theory	What methodological orientation was stated to underpin the study? *e.g. grounded theory, discourse analysis, ethnography, phenomenology, content analysis*	Phenomenological hermeneutical analysis.
Participant selection			
10.	Sampling	How were participants selected? *e.g. purposive, convenience, consecutive, snowball*	Purposive<br>More information is given in the method section.
11.	Method of approach	How were participants approached? *e.g. face-to-face, telephone, mail, email*	To recruit participants, information sheets about the study were distributed both physically and digitally. Relatives interested in participating contacted the first author via phone, e-mail, or left their phone number for the researcher to contact them.

			More information is given in the method section.
12.	Sample size	How many participants were in the study?	14 relatives participated.
13.	Non-participation	How many people refused to participate or dropped out? Reasons?	None
Setting			
14.	Setting of data collection	Where was the data collected? *e.g. home, clinic, workplace*	Twelve interviews were conducted face-to-face, and two of the interviews were conducted digitally.
15.	Presence of non-participants	Was anyone else present besides the participants and researchers?	No
16.	Description of sample	What are the important characteristics of the sample*? e.g. demographic data, date*	The sample consisted of both women and men, aged 32–85 years, who were either a spouse, partner, child, parent, or sibling of the deceased, from seven different municipalities. See Table 1 for the characteristics of the sample.
Data collection			
17.	Interview guide	Were questions, prompts, guides provided by the authors? Was it pilot tested?	A semi-structured interview guide was used. A pilot interview was held to evaluate the questions. See Table 2 for a description of the questions in the interview guide.
18.	Repeat interviews	Were repeat interviews carried out? If yes, how many?	No
19.	Audio/visual recording	Did the research use audio or visual recording to collect the data?	The interviews were audio-recorded.
20.	Field notes	Were field notes made during and/or after the interview or focus group?	Yes, during and directly after the interviews.
21.	Duration	What was the duration of the interviews or focus group?	The interviews lasted between 55 and 150 minutes, with an average time of 92 minutes.
22.	Data saturation	Was data saturation discussed?	Yes.<br>After 14 interviews, no new information seemed to emerge, and data analysis continued until consensus was reached.
23.	Transcripts returned	Were transcripts returned to participants for comment and/or correction?	No
**Domain 3: analysis and findings**			
Data analysis			
24.	Number of data coders	How many data coders coded the data?	EMM was the main coder of the data and made a preliminary analysis and discussed it with KSF, and then shared it with SP, TS and TL for further discussion.
25.	Description of the coding tree	Did authors provide a description of the coding tree?	No, but quotes are included in the results.
26.	Derivation of themes	Were themes identified in advance or derived from the data?	The themes were derived from the data.
27.	Software	What software, if applicable, was used to manage the data?	No

28.	Participant checking	Did participants provide feedback on the findings?	No
Reporting			
29.	Quotations presented	Were participant quotations presented to illustrate the themes / findings? Was each quotation identified? *e.g. participant number*	Quotations were presented to illustrate the findings. Each quotation is identified by participant number.
30.	Data and findings consistent	Was there consistency between the data presented and the findings?	Yes
31.	Clarity of major themes	Were major themes clearly presented in the findings?	Yes<br>.
32.	Clarity of minor themes	Is there a description of diverse cases or discussion of minor themes?	Yes
